# If Mismatch Negativity Is the Answer, What Is the Question? On the Nature of Predictive Coding Abnormalities in Psychosis

**DOI:** 10.1016/j.bpsgos.2024.100412

**Published:** 2025-01-15

**Authors:** Valentina Mancini, Matthew M. Nour

**Affiliations:** a Department of Psychiatry, University of Oxford, Oxford, United Kingdom; b Wellcome Centre for Integrative Neuroimaging, FMRIB, Nuffield Department of Clinical Neurosciences, University of Oxford, Oxford, United Kingdom; c Medical Research Council Brain Network Dynamics Unit, University of Oxford, Oxford, United Kingdom; d Max Planck University College London Centre for Computational Psychiatry and Ageing Research, London, United Kingdom

Positive psychotic symptoms, such as hallucinations and delusions, reflect profound disruptions in the way that the brain forms and updates beliefs about the world. Predictive coding theories posit that this process approximates Bayesian inference on the latent causes of sensory data; observations (likelihoods) are weighed against expectations (priors) to arrive at better beliefs (posterior probabilities) (1,2). Prediction errors signal the discrepancy between likelihoods and priors, guiding adaptive belief updating. Reflecting the brain’s hierarchical organization, beliefs become more abstract and spatiotemporally extended as we ascend a putative cortical processing hierarchy, analogous to the levels of abstraction inherent in comprehending a passage of text (letters < words < sentences < paragraphs < narrative events).

SEE CORRESPONDING ARTICLE NO. 100394

In psychosis, predictive coding accounts assert that abnormal prediction error–mediated belief updating underlies delusions and hallucinations, both construed as false inferences. While specific proposals differ in their details, a common theme implicates the arbitration between priors and likelihoods—a process termed “precision weighting”—at specific hierarchical levels. This miscalibration in turn results in cognitive and perceptual abnormalities either as a direct result of abnormal inference or secondary to compensatory neurocomputational mechanisms (2). This framework has been enormously influential in the computational psychiatry of psychosis. However, empirical progress has arguably been stymied by difficulties in formulating experimental tests. It can be hard to pin down a model with so many moving parts.

A common approach has been to use simple decision making or perceptual tasks, where experimenters are able to control the predictability of presented stimuli and, in some cases, infer participant beliefs through choice behavior. In a paradigmatic example, participants are presented with a sequence of identical auditory tones (standard tones) followed by the presentation of a deviant tone that differs on 1 auditory feature (e.g., duration, frequency). When conducted during electroencephalography, the unexpectedness of the deviant tone evokes an event-related potential that differs in amplitude from that evoked by the standard tone. This mismatch negativity (MMN) is hypothesized to reflect sensory prediction error processing. A reduction in MMN amplitude is one of the most replicated findings in people with schizophrenia (3), including those experiencing a first episode, broadly consistent with predictive coding accounts. However, there is inconsistent evidence for a correlation between MMN and positive symptoms, which raises questions about predictive coding accounts of positive symptoms. Is abnormal prediction error signaling necessary for the expression of hallucinations and delusions, or is it instead a more nonspecific disease marker in schizophrenia and related disorders?

**Figure 1:**
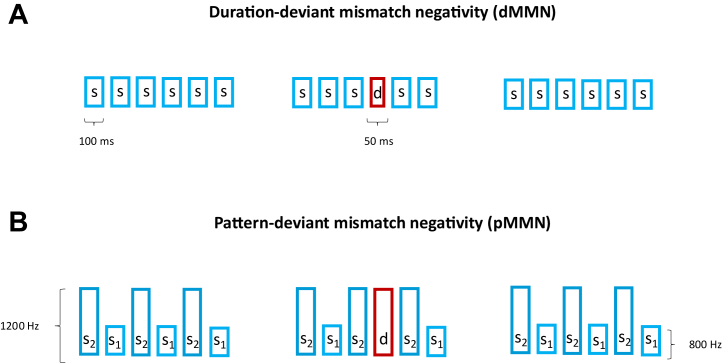
MMN tasks. **(A)** In the dMMN, a stimulus train consists of 6 auditory tones. Standard tones have a duration of 100 ms (87.5% of trials contain 6 standard tones), while deviant tones have a duration of 50 ms (12.5% of trials have a single deviant tone). **(B)** In the pMNN, a stimulus train consists of 6 auditory tones, which alternate between 2 frequencies (pitch, s_1_ = 800 Hz, s_2_ = 1200 Hz). Standard trials (87.5%) follow this alternation pattern, while deviant trials (12.5%) have a repeated *s*_2_ in the fourth position, thereby violating the alternation pattern.

Just published in *Biological Psychiatry: Global Open Science*, Erickson *et al.* (4) take steps to address this knowledge gap. They recruited 3 groups of participants: a group of people with a diagnosis of schizophrenia (PSZ, *n* = 56), a group of individuals who experienced frequent and intense hallucinations without functional impairment (nonclinical voice hearers [NCVHs], *n* = 34), and a group of control participants (*n* = 48). Each participant performed 2 MMN paradigms (order counterbalanced): first, a “duration-deviant MMN” similar to the one described above, wherein a deviation is defined according to low-level perceptual criterion (50 vs. 100 ms tone duration), and second, a “pattern MMN,” where deviance is defined on the basis of the relationships between pairs of tones (a higher-order construct hypothesized to be represented at deeper levels of a cortical processing hierarchy) (Figure 1). Consistent with the preattentive nature of the task, participants were not required to elicit a behavioral response and were instead instructed to watch a silent movie during tone presentation.

Consistent with the literature, PSZ exhibited a reduction in both MMN variants compared with control participants, with the most convincing effect seen in the low-level duration-deviant variant. By contrast, NCVHs, who reported weekly hallucinations of a severity comparable to PSZ, showed no such MMN blunting (4). Moreover, there was no relationship between MMN reductions and symptom severity either in the PSZ or the NCVH group.

This pattern argues against a simple hypothesis that predictive coding abnormalities, as measured by blunted MMN responses, drive the expression of established hallucinations either in people with severe mental illness or people with minimal functional and cognitive impairment. This conjecture is supported by alternative lines of evidence: MMN abnormalities are not strongly ameliorated by dopamine D_2/3_ receptor antagonist medications that are routinely used to treat positive symptoms (5). However, the MMN is modulated by glutamatergic and muscarinic pharmacological perturbations considered to be relevant to the pathophysiology of schizophrenia (6).

Therefore, one interpretation is that the MMN does not track processes relevant to hallucinations, but rather indexes more nonspecific factors that characterize the neurocognitive profiles of PSZ (and potentially, people exhibiting similar degrees of functional impairment in the context of severe mental illness). The authors highlight important considerations that complicate this interpretation, including whether the mechanisms of symptom emergence differ from those that underlie maintenance, and the questionable assumption that the phenomenology of hallucinations in NCVHs and PSZ exist on a meaningful continuum.

What does this mean for the predictive coding account of psychosis more broadly? A conservative possibility, highlighted by the authors, is that classical MMN paradigms may simply be ill equipped to index the aspects of predictive coding most relevant to psychosis. The duration-deviant MMN employed by Erickson *et al.* (4) was designed to index sensory prediction errors that act on fast time scales in early cortical processing layers. The pattern MMN, while indexing prediction errors at a higher level of abstraction, is also a preattentive sensory paradigm defined on low-level sensory features. Moreover, both paradigms engage only a single level of a predictive coding hierarchy.

Other work has demonstrated the power of examining beliefs at multiple levels of a processing hierarchy. One landmark study used an audiovisual conditioning paradigm and modeled behavior using a hierarchical Gaussian filter (a Bayesian model) capable of inferring beliefs at several hierarchical levels: low-level perceptual beliefs, beliefs about auditory-visual associations, beliefs about the trial-by-trial volatility of these associations, and the weighting of priors relative to observations. When they compared 4 groups—control participants, NCVHs, and psychotic individuals with and without hallucinations—the authors found that hallucinations were linked in all groups to stronger priors on lower-level perceptual beliefs, while psychosis was characterized by reduced evolution of beliefs on volatility, regardless of hallucination status (7).

Notwithstanding these notable findings, it is still true that most experimental paradigms in computational psychiatry operate by abstracting out the key task features under investigation and bringing these under tight experimental control. Such experimental abstraction is a double-edged sword: The gains afforded by increased experimental control may be outweighed by a loss of ecological (and thus, clinical) validity. Delusions and hallucinations are invariably more complex and abstract in their content than the identity of a single tone (or even the identity of a pair of tones). Accordingly, it is likely that the underlying neural representations of these more complex and temporally extended beliefs reside at far deeper levels of a cortical processing hierarchy. The default mode network, which sits at the apex of such a hierarchy (8), has been shown to represent narrative schemas during naturalistic viewing paradigms, and to encode structural priors regarding how the stream of sensory data segments into meaningful events extending over seconds (9). Structured representations at these higher levels of abstraction have come to be termed “cognitive maps” (10). Tracking predictive coding abnormalities relevant to these representations is likely to require new tasks and analytic approaches capable of indexing the evolution and violation of expectations in more naturalistic—and thus more clinically valid—contexts.

However, it is important to acknowledge the risk of adding epicycles to epicycles. One concern about predictive coding theories is their flexibility: with so many moving parts, there is always the potential to modify the model to accommodate conflicting evidence. This adaptability, while theoretically valuable, runs the risk of diluting the theory’s potential explanatory power. The study by Erickson *et al.* (4) offers a valuable contribution in this context. Challenging the presumed tight relationship between MMN and hallucinations prompts a necessary reexamination of the experimental paradigms used to index prediction error–mediated processing relevant to positive symptoms. Such work is essential for refining the predictive coding framework and ensuring that it generates testable, empirically grounded hypotheses in the future.
